# Complex Chemosensory Control of Female Reproductive Behaviors

**DOI:** 10.1371/journal.pone.0090368

**Published:** 2014-02-28

**Authors:** Eleanor J. Fraser, Nirao M. Shah

**Affiliations:** Program in Genetics, Department of Anatomy, University of California San Francisco, San Francisco, California, United States of America; The University of Tokyo, Japan

## Abstract

Olfaction exerts a profound influence on reproductive physiology and behavior in many animals, including rodents. Odors are recognized by sensory neurons residing in the main olfactory epithelium (MOE) and the vomeronasal organ (VNO) in mice and many other vertebrates. The relative contributions of the MOE and VNO in the display of female behaviors are not well understood. Mice null for *Cnga2* or *Trpc2* essentially lack odor-evoked activity in the MOE and VNO, respectively. Using females mutant for one or both of *Cnga2* and *Trpc2*, we find that maternal care is differentially regulated by the MOE and VNO: retrieval of wandering pups requires the MOE and is regulated redundantly by the VNO whereas maternal aggression requires both sensory epithelia to be functional. Female sexual receptivity appears to be regulated by both the MOE and VNO. *Trpc2* null females have previously been shown to display male-type mounting towards other males. Remarkably, we find that females double mutant for *Cnga2* and *Trpc2* continue to mount other males, indicating that the disinhibition of male-type sexual displays observed in *Trpc2* null females does not require chemosensory input from a functional MOE. Taken together, our findings reveal a previously unappreciated complexity in the chemosensory control of reproductive behaviors in the female mouse.

## Introduction

Many animals utilize olfaction as their primary sensory modality to recognize mates, food, and predators. A special class of species-specific odorants known as pheromones is secreted by animals to signal social and reproductive status to conspecifics (other members of the species) [1], [2]. Such pheromones can elicit diverse physiological responses such as modulation of the length of the estrous cycle as well as behavioral displays such as mating, aggression, and nursing [3]. Pheromones are recognized by sensory neurons residing in the MOE and the VNO in mice and many other animals [4]. These two epithelia utilize distinct signaling pathways to transduce odor recognition into neural activity, thereby permitting genetic analysis of the relative importance of the MOE and the VNO in reproductive physiology and behavior. Such genetic studies in male mice have shown that both the MOE and VNO are essential for aggressive displays [5]–[8]. These studies show that control of sexual behavior in males is more complex, such that the MOE is required for the display of this behavior whereas the VNO functions to suppress the display of this behavior toward other males [5]–[9]. Although pheromone recognition is also important for normal female reproductive behaviors [3], the relative genetic contribution of signaling via the MOE and the VNO in these processes remains to be determined.

*Cnga2* and *Trpc2* encode cation channels that are expressed in an essentially mutually exclusive manner in the MOE and VNO, respectively [5], [10], [11]. Moreover, Cnga2 and Trpc2 are essential for normal odor-evoked neural activity in these two sensory epithelia [6], [7], [12]. We have analyzed females mutant for one or both of Cnga2 and Trpc2 in order to determine the relative contribution of MOE and VNO signaling to female reproductive behaviors. We find that Cnga2 and Trpc2 appear not to be required for the estrous cycle, but that both play important roles in female sexual behavior, maternal care, and defense of young.

## Materials and Methods

### Animals

Adult mice (>8 weeks of age) were used for all studies unless otherwise noted. The mice were housed in a barrier facility in ventilated cage rack systems with Paperchip bedding (Sheppard Specialty Paper) under a 12∶12 light∶dark cycle, group-housed by sex at weaning, and food and water were provided ad libitum. Mice bearing null alleles of *Cnga2* and *Trpc2* used in this study have been described previously [7], [12], and these were maintained on a mixed 129/Sv and C57Bl/6J background. *Trpc2^–/–^* females were obtained from crosses in which one or both parents were homozygous for the *Trpc2* allele; *Cnga2^–/–^* females were obtained by breeding *Cnga2^−/+^* females to *Cnga2^–/Y^* males; double mutant *Cnga2^–/–^*, *Trpc2^–/–^* females were obtained with the breeding strategy described above for *Cnga2^–/–^* females in a homozygous Trpc2 null background. Control females were age-matched *Cnga2^−/+^*, *Trpc2^−/+^*, or WT females obtained from crosses used to generate animals heterozygous or homozygous null for Cnga2 or Trpc2. Mice null for Cnga2 have difficulty feeding early in life, and they were raised as described earlier [5], [12]. All in vivo experiments were in accordance with the ARRIVE guidelines. All studies involving animals were approved by the Institutional Animal Care and Use Committee at the University of California San Francisco.

### Behavior

Behavioral testing was initiated ≥1 hour after onset of the dark cycle, and the behaviors were recorded and analyzed as described previously [13]. Briefly, female sexual receptivity was tested in adult females that had been ovariectomized and allowed >1 week of recovery following surgery. Estrus was induced with hormone priming exactly as described earlier [13] (10 µg and 5 µg of 17 β-estradiol benzoate dissolved in sesame oil at 0.1 mg/mL on days -2 and -1 and 50 µg of progesterone dissolved in sesame oil at 1 mg/mL on the day of testing), and the female was inserted into the cage of a sexually experienced WT male for 30 min. Each female was tested 3–4 times for sexual receptivity, with each test separated by ≥7 days. The sexual receptivity index is the fraction of mounts that the female permits to proceed to intromission.

To test for nest building, pup retrieval, and maternal aggression, females were co-housed with males but singly housed prior to parturition. Singly housed pregnant females were transferred to a fresh cage, provided with Nestlets (Ancare), and the quality of the nest was assessed 24 hours later. These females were subsequently allowed to deliver their litter, and their pup retrieval ability was tested. Cnga2 is X-linked and therefore all male progeny of Cnga2 null females are anosmic and at a competitive disadvantage at successful suckling unless the litter size is small or experimentally reduced. Since we wished to compare maternal behaviors across different genotypes, we did not want to selectively trim litters of Cnga2 null or Cnga2 and Trpc2 double mutant females. Accordingly, we trimmed litters from females of each genotype within 6–12 hours after birth by eliminating Cnga2 mutant males (as revealed by the absence of the characteristic abdominal milk spot) while still retaining a litter size of 3–4 pups to ensure that the dam continued to lactate and exhibit maternal behaviors. At 2, 4, and 6 days (±1) after parturition, the dam was removed briefly from her cage, and three of her pups were placed in the cage corner farthest from the nest. The dam was returned to her cage and pup retrieval behavior was observed for 15 min. Subsequent to these pup retrieval assays, we tested the dam for maternal aggression at 8, 10, and 12 days (±1) after parturition. The pups were removed from the cage, and a group-housed WT male intruder was inserted into the cage for 15 min. At the end of the testing period, the male was removed from the cage, and the pups were returned to the mother.

To test for male-type mating behavior, adult sexually naïve females were singly housed for 2–7 days in a fresh cage. A WT group-housed vasectomized male intruder was inserted for 15 min into the female's cage, and each female was tested 3 times. The females were always exposed to males they had not encountered previously, and each assay was separated by ≥2 days.

### Estrous cycle

The stage of the estrous cycle was assessed by daily vaginal cytology for 5 weeks in group-housed females (3–5 females per cage). The cytological characteristics of the vaginal smear were examined with brightfield optics as described previously [13], [14]. The length of an estrous cycle was defined as the number of days between two non-consecutive days during which estrus cytology was observed.

### Statistical Analysis

Quantification of behavioral and estrous cycle data was performed blind to genotype. We performed the following tests of statistical significance. Categorical data was analyzed using a Fisher's exact test with a Bonferroni correction for multiple comparisons. Continuous data from multiple assays per animal was first averaged within each animal and then compared across groups. Data for a behavior or estrous cycle includes only assays in which the behavior (or estrous cycle) occurred. For continuous data, we first tested the distribution of the data with Lilliefors' goodness-of-fit test of normality. Data not violating the assumption of normality was analyzed using parametric tests (ANOVA for ≥3 groups with Tukey post-hoc test, Student's t test for 2 groups) whereas data with a non-normal distribution was analyzed with the non-parametric Kruskal-Wallis (with Tukey's post-hoc test) or Kolmogorov-Smirnov test. We used an alpha level of 0.05 for all statistical tests.

## Results

### Signaling via the MOE and VNO is required for female sexual behavior but not estrous cyclicity

**Figure 1:**
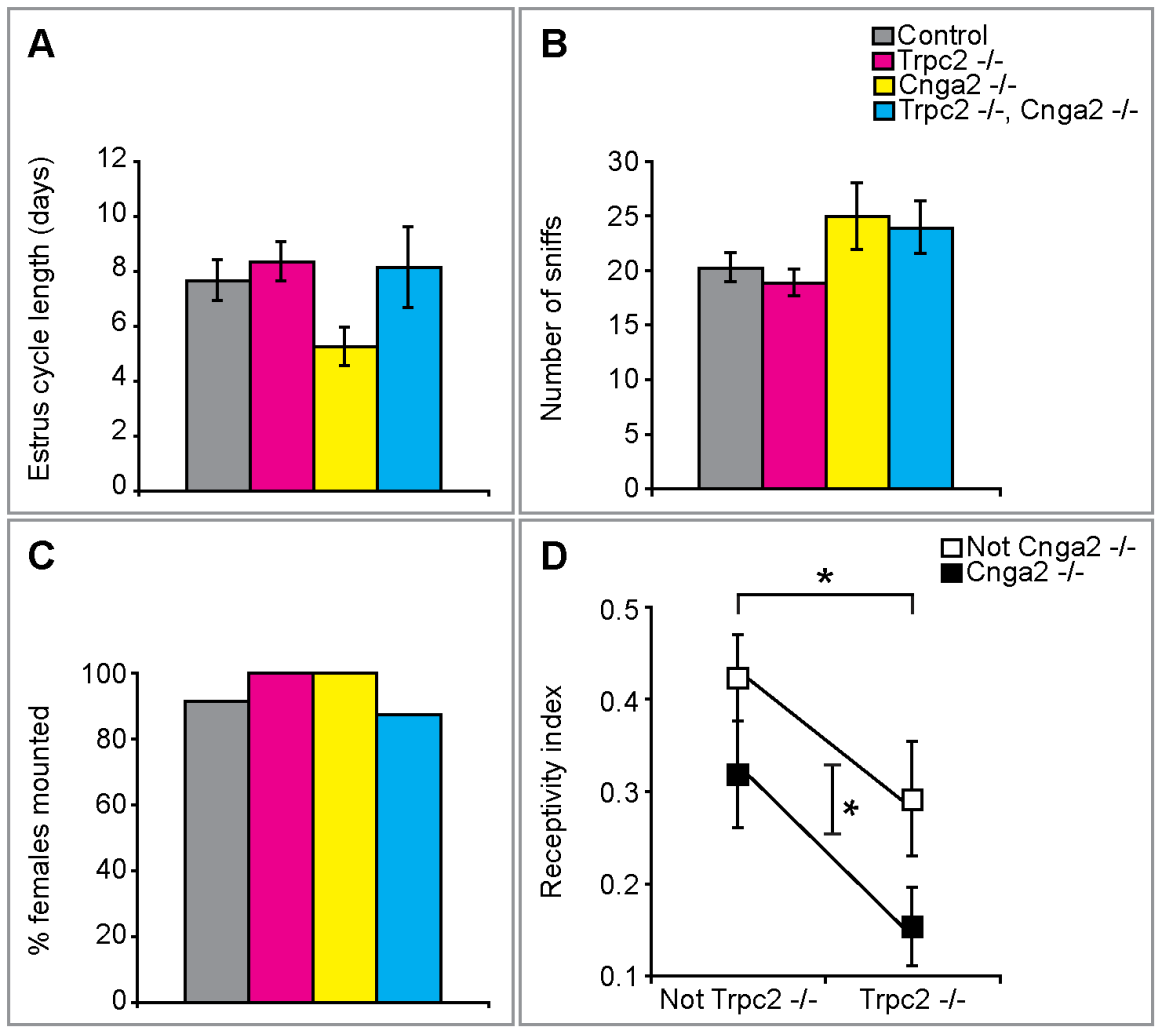
Odor-evoked activity in the MOE and VNO is required for female sexual receptivity. (A) No difference between single or double mutant and control females in the length of the estrus cycle. (B) Males showed comparable frequency of anogenital chemoinvestigation (sniffing) to females of all genotypes. (C) A majority of females of all genotypes were mounted by WT males in ≥50% of assays. (D) Chemosensory control of female sexual receptivity. There was a significant effect on receptivity from both *Cnga2* and *Trpc2*, with no interaction between the two, resulting in a significant decrease in sexual receptivity in *Cnga2^-/-^*, *Trpc2^-/-^* double mutant females. Two-way ANOVA: *Cnga2 F*(1,56)  = 4.05, p = 0.049; *Trpc2 F*(1,56)  = 6.03, p = 0.017; interaction *F*(1,56)  = 0.077, p = 0.782. Mean ± SEM; *p<0.05; N≥8/cohort.

Pheromones have been implicated in regulating the length of the estrous cycle as well as sexually receptive behavior to males [3], [15], [16]. In initial studies, we assessed the estrous cycle in females lacking *Cnga2* (*Cnga2^–/–^*), *Trpc2* (*Trpc2 ^–/–^*), or both (*Cnga2^–/–^*, *Trpc2^–/–^*). Strikingly, we observed that the length of the estrous cycle in females group-housed by sex since weaning was similar between Cnga2 and Trpc2 single or double mutants and control females (Figure 1A). Although Cnga2 null females appeared to have a shorter estrous cycle compared to controls, this difference was not statistically significant. However, we cannot exclude a subtle effect of Cnga2 on estrous cycle length, an effect that could be investigated in future studies. We did not investigate the effects of male pheromones or female housing density on estrous cycle length [3], and it is possible that such cues require odor-evoked signaling via Cnga2 or Trpc2 to modulate the estrous cycle. Lesioning the MOE or the VNO reveals a requirement for one or both epithelia in sexual receptivity, depending on the species [15], [17], [18]. We therefore wished to assess genetically whether signaling via the MOE or VNO was required for sexual receptivity in mice. Although the estrous cycle appears unaffected in mutant females, we cannot exclude subtle changes during estrus that would affect mating behavior. Accordingly, we ovariectomized adult females, allowed them to recover from this surgery, and hormonally primed them to be in estrus prior to testing for sexual receptivity with wildtype (WT) sexually experienced males [19]. Sexually receptive female mice allow a male to approach them and permit mounting to proceed to intromission (penetration) [20]. Females of all genotypes appeared equivalently attractive to the WT stud males, as revealed by the males' interest in chemoinvestigating them (Figure 1B). Consistent with this notion, the WT stud males also mounted females of all genotypes in a comparable percent of assays (Figure 1C), and the males mounted mutant females at least as frequently as control females (Figure S1). Nevertheless, we observed that Trpc2 as well as Cnga2 null females allowed fewer mounts to proceed to intromission, indicative of reduced sexual receptivity (Figure 1D).

Despite the diminution in sexual behavior in females lacking either Cnga2 or Trpc2, these mice were nevertheless receptive to WT males. Such residual receptivity could reflect compensatory mechanisms triggered in response to deficits in MOE or VNO signaling such that females unable to detect odors via both epithelia would be completely unreceptive. We therefore tested sexual behavior in *Cnga2^–/–^*, *Trpc2^–/–^* double mutant females. We observed that although there was a further reduction in receptivity in these double mutants, this reduction reflected an additive rather than a synergistic effect of deleting both Cnga2 and Trpc2 (Figure 1D). Thus, the MOE and VNO play a non-redundant role in influencing female sexual behavior. However, female sexual behavior is not reliant on chemosensory input, because even Cnga2 and Trpc2 double mutants exhibited some degree of sexual receptivity. This conclusion is further strengthened by our observation that upon chronic co-housing with a male, control as well as single or double mutant females yielded litters at a similar frequency (Figure S2A). Taken together, our studies demonstrate the surprising finding that a functional VNO and MOE are important, but not essential, for the display of female sexual behavior.

### Both the MOE and VNO contribute to maternal care and defense of pups

We assessed the requirement of Cnga2 or Trpc2 in various elements of maternal behaviors. We chronically co-housed experimental females with control males and subsequently housed the females individually prior to parturition. Provision of nesting material to such females elicited significant nest-building that was comparable across all genotypes (Figure S2B, C). Upon parturition, dams of all genotypes ate their afterbirths, cleaned the pups, and gathered them in the nest in a comparable manner. Thus these early components of maternal behavior, including nest building, placentophagia, and initial care of newborns, are independent of Cnga2 and Trpc2 signaling.

**Figure 2:**
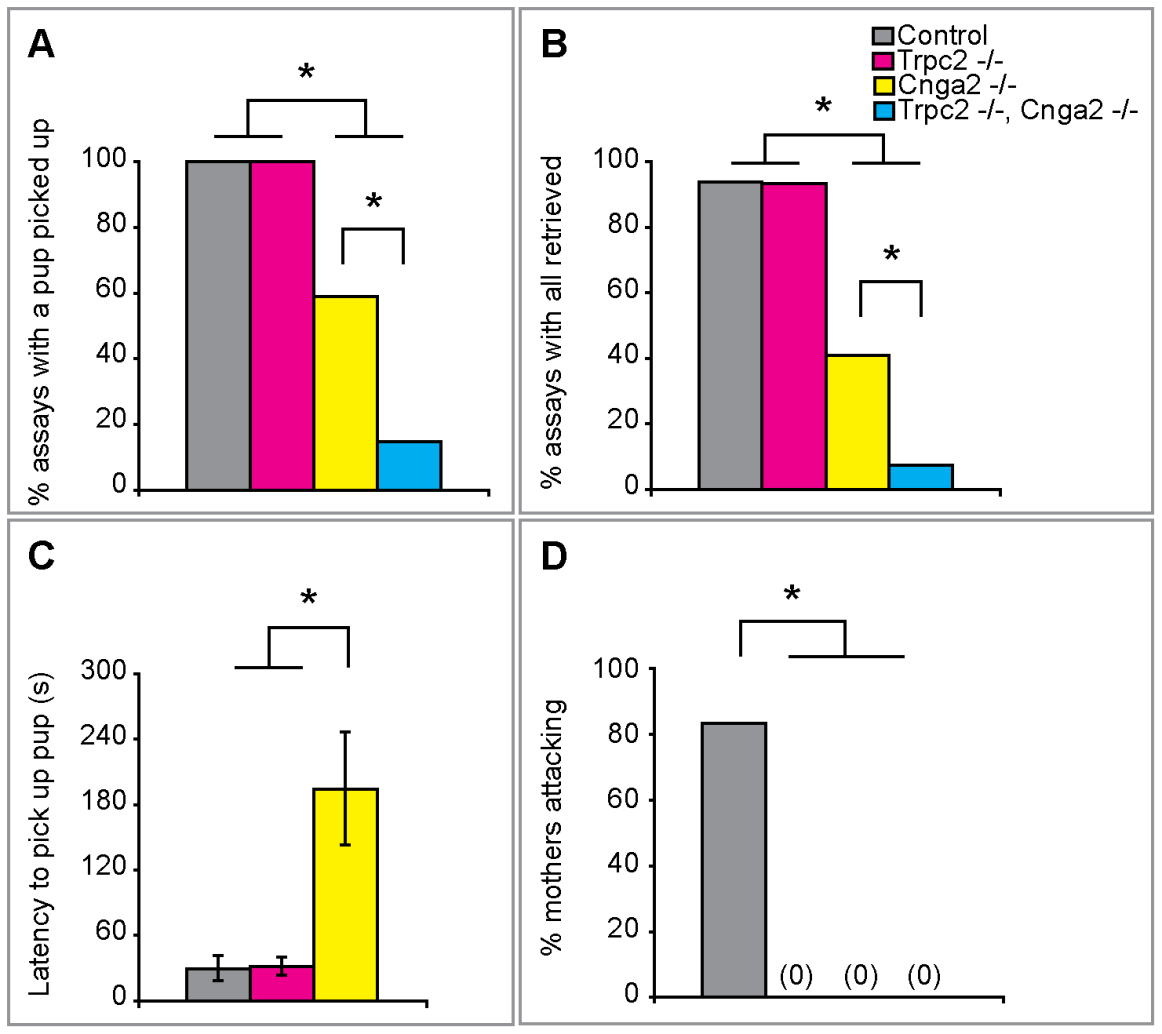
Odor-evoked activity in the MOE and VNO is required for maternal behaviors. (A, B) *Cnga2^-/-^* dams were less likely either to pick up a pup or to return all pups to the nest. The double mutant showed an even greater reduction in retrieval behavior. (C) *Cnga2^-/-^* females were slower to begin retrieval of pups than either controls or *Trpc2^-/-^* females. (Double mutant data not shown because only 2 assays included retrieval.) Kruskal-Wallis: χ^2^(2,24)  = 9.71, p = 0.008 for the overall comparison. (D) The absence of Cnga2 or Trpc2 abolished maternal aggression. Mean ± SEM; *p<0.05. (a–c) N≥8/cohort; (d) N≥4/cohort.

Mouse pups can crawl away from their nest and dams efficiently retrieve them back to the nest, presumably to provide nutrition and prevent hypothermia and predation. In order to test such pup retrieval, we moved the pups to a cage corner farthest away from the nest and observed the nursing females for 15 min for pup retrieval. In these tests, *Cnga2^–/–^* and *Cnga2^–/–^*, *Trpc2^–/–^* mothers showed a significant diminution in pup retrieval compared to WT or *Trpc2^–/–^* mothers such that in >66% of all assays they failed to retrieve all pups back to the nest (Figure 2A-C). Such failure to bring pups back to the nest was not simply because these females did not venture out from their nest; on the contrary, their locomotor activity was comparable to WT mothers (Figure S3), and they often walked very close to or, on occasion, even over the pups without picking them up (Movies S1, S2). Surprisingly, the *Cnga2^–/–^*, *Trpc2^–/–^* mothers showed significantly diminished pup retrieval compared to *Cnga2^–/–^* females even though *Trpc2^–/–^* females were comparable to WT mothers in this test (Figure 2A, B). Our findings show that the MOE is essential for pup retrieval whereas the contribution of the VNO to this behavior is redundant to that of the MOE.

Nursing mice will attack unfamiliar intruders in their cage because such intruders can display infanticide or aggression toward pups [21]. Trpc2-mediated VNO signaling is essential for the normal display of such maternal aggression [7], [22]. We asked whether Cnga2-mediated signaling was also required for maternal aggression. We therefore inserted a WT intruder male into the cage of a nursing *Cnga2^–/–^* mouse and tested for aggression toward the male. In contrast to control females, we observed that Cnga2 mutant or Cnga2 and Trpc2 double mutant mothers never initiated attacks toward the intruder (Figure 2D). Thus, and in accord with prior genetic studies [7], [22], [23], both the MOE and VNO are individually essential for maternal aggression.

### Females display male-type sexual behavior in the absence of signaling via the MOE or the VNO

**Figure 3:**
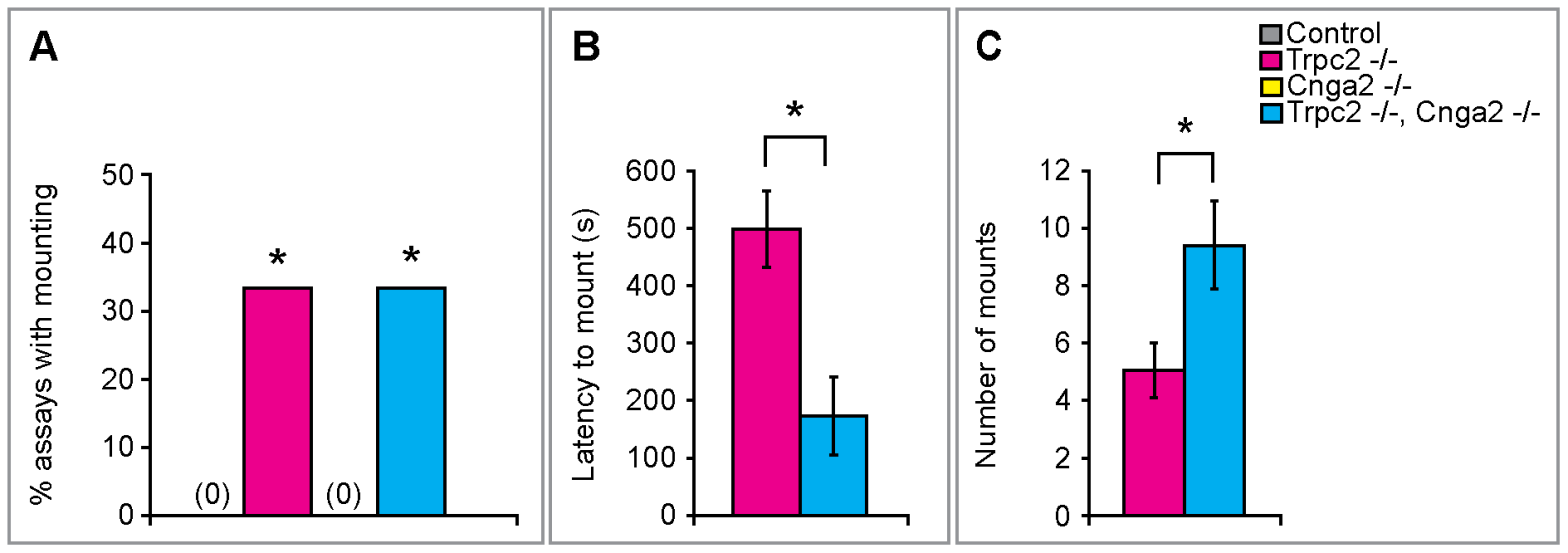
Odor-evoked activity in the MOE is not required for male-typical sexual displays. (A) *Trpc2^-/-^* and *Cnga2^-/-^*, *Trpc2^-/-^* females were equally likely to mount a male and significantly different from control females who never mounted males. (B) *Cnga2^-/-^*, *Trpc2^-/-^* females mount males earlier than *Trpc2^-/-^* females. t-test *t*(13)  = −2.543, p = 0.025. (C) *Cnga2^-/-^*, *Trpc2^-/-^* females mount males more often than *Trpc2^-/-^* females. t-test *t*(13)  = 3.28, p = 0.006. Mean ± SEM. *p<0.05. N≥11/cohort.

WT females can display male-type mounting behavior toward females, but they rarely mount WT males [24]–[27]. However, *Trpc2^–/–^* females do exhibit male-type mounting toward males [26]. Such male-typical mounting likely reflects the disabled VNO sensory neuron signaling engendered by loss of Trpc2. Consistent with this notion, Trpc2 is not expressed in the adult brain [10]; it is nevertheless possible that low-level adult or transient developmental expression of Trpc2 in the brain can alter the function of neural circuits that regulate male sexual displays. In any event, Cnga2 is required for the display of male-typical mating [5], [9], and we wished to test whether Cnga2-mediated signaling was essential for this behavior in Trpc2 null females. Accordingly, we tested singly housed WT, *Cnga2^–/–^*, *Trpc2^–/–^*, and *Cnga2^–/–^*, *Trpc2^–/–^* females for the display of male sexual behavior toward a WT male intruder in their cage. As expected, neither the WT nor the Cnga2 mutant females exhibited any mounting behavior in these assays whereas Trpc2 mutant females did mount males (Figure 3A). These Trpc2 null females mounted males at a slightly lower frequency compared to that reported previously [26], likely reflecting the differences in the experimental setup. Whereas the previous study utilized castrate or bulbectomized males as intruders, we used gonadally intact males with a functional chemosensory system. Since our intruder males often mounted the resident females, it is even more striking that we observed females mounting such intact males. Dramatically, females doubly mutant for Cnga2 and Trpc2 mounted males with a probability equivalent to Trpc2 mutant females (Figure 3A). Moreover, the double mutants mounted males more and faster than Trpc2 mutants (Figure 3B, C). Thus, we find that a functioning MOE is not required for male-type mating behavior in females, and in fact, the MOE might sense cues emanating from males that inhibit male-type mounting by females.

## Discussion

We have analyzed the genetic requirement for odor-evoked signaling in the MOE and VNO for female reproductive behaviors in mice. We find that the MOE and VNO are required for the normal display of sexual receptivity and maternal aggression in a non-redundant manner. By contrast, a functional MOE can compensate for the disabled VNO signaling in Trpc2 mutants during pup retrieval. Strikingly, we find that male-type sexual behavior can be displayed by females independent of normal chemosensory signaling via the MOE and VNO.

The expression of Trpc2 is largely restricted to the VNO and is required for normal olfactory-ligand evoked signaling in VNO neurons [6], [7], [10]. Thus, our findings demonstrate that the VNO plays an important role in female sexual behavior and maternal care. Moreover they corroborate earlier studies showing reduced sexual behavior in Trpc2 null females and in females with surgical removal of the adult VNO [17], [28]. A previous study did not report reduced female sexual receptivity in Trpc2 null mice [26], but this is likely due to differences in the setup of the behavioral assays. Indeed, we, and other groups who reported diminution of female sexual receptivity in mice with disabled VNO signaling, used hormonally primed estrus females in one-to-one encounters with wildtype males. By contrast, no diminution of sexual receptivity was observed in gonadally intact group housed Trpc2 null females that encountered wildtype males. In any event, the importance of the VNO in female sexual behavior is underscored by the finding that a male-derived pheromone specifically activates a VNO chemoreceptor that is essential for the high wildtype levels of female sexual receptivity [28]. Our findings are in agreement with prior work showing that Trpc2 null females or females surgically deprived of the VNO display a profound reduction of maternal aggression [7], [22], [29]. Previous studies with Trpc2 null nursing females suggested that these mice exhibited a shorter latency to crouch over their nests but spent less time crouching and nest building [26], [30]. Our findings significantly extend these observations by showing that the VNO is important in retrieving pups back to the nest in the absence of a functional MOE, but it is not essential for building a nest pre-partum. Thus, distinct neural pathways may be utilized to build a nest pre-partum and post-partum.

We find that Cnga2 mutant females exhibit significant deficits in sexual receptivity, pup retrieval, and maternal aggression. Although Cnga2 is expressed in the brain, these behavioral deficits are unlikely to reflect a central rather than sensory requirement of Cnga2 [31]. Indeed, similar phenotypes have been observed by complementary studies that disrupt MOE function [18], [23], [32], and in fact our studies significantly extend these previous observations. We find that Cnga2 mutant females build a nest and, in the absence of experimental manipulations, can produce litters that survive to weaning (Figure S2D). This is in contrast to females mutant for other components of odor-evoked signal transduction, G_olf_ or adenylate cyclase III (AC3), whose litters do not survive to weaning [23], [33]. The more severe deficits in G_olf_ and AC3 mutants may reflect their expression in the brain and other tissues; consistent with this notion, G_olf_ mutants display increased locomotor activity, possibly due to G_olf_ expression in the basal ganglia, and AC3 null mice are obese [18], [34]. Alternatively, this difference may reflect the possibility that mice mutant for G_olf_ or AC3 have a more severe deficit in odor-evoked signal transduction compared to Cnga2 mutants. In fact, MOE neurons from Cnga2 null mice can respond to some odorants, suggesting residual olfactory signaling in these mutants [35], [36]. Importantly, it remains to be determined whether mice null for G_olf_ or AC3 also respond to these odors.

We find that both the MOE and VNO play important roles in female sexual receptivity, maternal aggression, and care of young. Genetic disabling of the MOE or VNO in male mice reveals a similar dual requirement for the display of intermale aggression [5]–[7]. By contrast, the control of male sexual behavior is more complex such that the MOE is essential for sexual displays in males whereas the VNO is required to restrict such displays to mice of the opposite sex [5]–[7], [9]. Such dual contributions from the MOE and VNO for the normal display of innate social displays may reflect evolutionary selection for redundancy in the control of behaviors critical for reproductive success. Alternatively, mice may encounter diverse salient pheromonal cues that activate the VNO or the MOE in different settings in nature.

It is surprising that females mutant for both MOE and VNO signaling continue to display male-typical sexual displays toward males. The display of male-type mating in these females may rely on Cnga2- and Trpc2-independent activation of MOE or VNO sensory neurons. Consistent with this notion, recent studies suggest that VNO neurons can respond to chemosensory cues in Trpc2 null mice [37], [38]; similarly some odors can elicit neural activity in a small subset of MOE sensory neurons independent of Cnga2 [35], [36]. Alternatively, our observation are consistent with the possibility that the disinhibition of male-pattern mating observed in Trpc2 mutants is independent of additional chemosensory input. In summary, our results reveal a dual requirement for the MOE and VNO in female reproductive behaviors and show that male-pattern mating can be elicited in the absence of Cnga2 and Trpc2 signaling.

## Supporting Information

Figure S1 **Decreased sexual receptivity of Cnga2 or Trpc2 null females is not a consequence of reduced mounting by WT males.** (A) WT males mount all females ≥10 mounts/assay on average. Cnga2 null females are mounted more times than Trpc2 null or control females. Kruskal-Wallis: χ^2^(3,56)  = 13.5, p = 0.007. (B) WT males mount all females within the first 400 s of an assay on average, which lasts for 1800 s. Cnga2 null females are mounted faster than control females. One-Way ANOVA: F(3,56)  = 3.08, p = 0.035. Mean ± SEM; *p<0.05; N≥8/cohort.
(PDF)

Figure S2 **Cnga2 and Trpc2 are not required for fertility, litter survival, or nest building.** (A) When co-housed with a male for at least 1 week, females of all genotypes produced litters within 26 days of co-housing. (B) Females of all genotypes built nests of equivalent quality as assessed by the presence of walls and a roof. (C) Females of all genotypes utilized comparable amount of nest material to construct their nests. (D) Comparable survival of litters delivered to females of all genotypes. Mean ± SEM. N≥7/cohort.
(PDF)

Figure S3 **Failure to retrieve pups in** ***Cnga2^-/-^*** **and** ***Cnga2^-/-^*** **,** ***Trpc2^-/-^*** **dams is not due to decreased locomotor activity.** Number of midline crosses during pup retrieval assays in olfactory mutants is comparable to controls. Mean ± SEM. N≥5/cohort.
(PDF)

Movie S1 **A control mother is shown retrieving her pups back to the nest.**
(MOV)

Movie S2 **A** ***Cnga2^–/–^*** **mother approaches but does not retrieve her pups back to the nest.**
(MOV)

## References

[1] Karlson P, Lüscher M (1959) ‘Pheromones’: a new term for a class of biologically active substances. Nature 183: 55–56. 13622694 10.1038/183055a0

[2] Stowers L, Marton TF (2005) What is a pheromone? Mammalian pheromones reconsidered. Neuron 46: 699–702. 15924856 10.1016/j.neuron.2005.04.032

[3] Halpern M (1987) The organization and function of the vomeronasal system. Annu Rev Neurosci 10: 325–362. 3032065 10.1146/annurev.ne.10.030187.001545

[4] Dulac C, Wagner S (2006) Genetic analysis of brain circuits underlying pheromone signaling. Annu Rev Genet 40: 449–467. 16953793 10.1146/annurev.genet.39.073003.093937

[5] Mandiyan VS, Coats JK, Shah NM (2005) Deficits in sexual and aggressive behaviors in Cnga2 mutant mice. Nat Neurosci 8: 1660–1662. 16261133 10.1038/nn1589

[6] Stowers L, Holy TE, Meister M, Dulac C, Koentges G (2002) Loss of sex discrimination and male-male aggression in mice deficient for TRP2. Science 295: 1493–1500. 11823606 10.1126/science.1069259

[7] Leypold BG, Yu CR, Leinders-Zufall T, Kim MM, Zufall F, et al (2002) Altered sexual and social behaviors in trp2 mutant mice. Proc Natl Acad Sci U S A 99: 6376–6381. 11972034 10.1073/pnas.082127599 PMC122956

[8] Wang Z, Sindreu CB, Li V, Nudelman A, Chan GC-K, et al (2006) Pheromone Detection in Male Mice Depends on Signaling through the Type 3 Adenylyl Cyclase in the Main Olfactory Epithelium. J Neurosci 26: 7375–7379. 16837584 10.1523/JNEUROSCI.1967-06.2006 PMC6674185

[9] Yoon H, Enquist LW, Dulac C (2005) Olfactory inputs to hypothalamic neurons controlling reproduction and fertility. Cell 123: 669–682. 16290037 10.1016/j.cell.2005.08.039

[10] Liman ER, Corey DP, Dulac C (1999) TRP2: a candidate transduction channel for mammalian pheromone sensory signaling. Proc Natl Acad Sci U S A 96: 5791–5796. 10318963 10.1073/pnas.96.10.5791 PMC21939

[11] Berghard A, Buck LB, Liman ER (1996) Evidence for distinct signaling mechanisms in two mammalian olfactory sense organs. Proc Natl Acad Sci U S A 93: 2365–2369. 8637879 10.1073/pnas.93.6.2365 PMC39802

[12] Brunet LJ, Gold GH, Ngai J (1996) General anosmia caused by a targeted disruption of the mouse olfactory cyclic nucleotide-gated cation channel. Neuron 17: 681–693. 8893025 10.1016/s0896-6273(00)80200-7

[13] Xu X, Coats JK, Yang CF, Wang A, Ahmed OM, et al (2012) Modular genetic control of sexually dimorphic behaviors. Cell 148: 596–607. 22304924 10.1016/j.cell.2011.12.018 PMC3326403

[14] Byers SL, Wiles MV, Dunn SL, Taft RA (2012) Mouse estrous cycle identification tool and images. PloS One 7: e35538. 22514749 10.1371/journal.pone.0035538 PMC3325956

[15] Dorries KM, Adkins-Regan E, Halpern BP (1997) Sensitivity and behavioral responses to the pheromone androstenone are not mediated by the vomeronasal organ in domestic pigs. Brain Behav Evol 49: 53–62. 8980852 10.1159/000112981

[16] Champlin AK (1971) Suppression of oestrus in grouped mice: the effects of various densities and the possible nature of the stimulus. J Reprod Fertil 27: 233–241. 5166413 10.1530/jrf.0.0270233

[17] Keller M, Pierman S, Douhard Q, Baum MJ, Bakker J (2006) The vomeronasal organ is required for the expression of lordosis behaviour, but not sex discrimination in female mice. Eur J Neurosci 23: 521–530. 16420459 10.1111/j.1460-9568.2005.04589.x PMC2266683

[18] Keller M, Douhard Q, Baum MJ, Bakker J (2006) Destruction of the main olfactory epithelium reduces female sexual behavior and olfactory investigation in female mice. Chem Senses 31: 315–323. 16484502 10.1093/chemse/bjj035 PMC2263131

[19] Ring JR (1944) The estrogen-progesterone induction of sexual receptivity in the spayed female mouse. Endocrinology 34: 269–275.

[20] McGill TE (1962) Sexual behavior in three inbred strains of mice. Behavior 19: 341–350.

[21] Gandelman R (1972) Mice: postpartum aggression elicited by the presence of an intruder. Horm Behav 3: 23–28. 4681734 10.1016/0018-506x(72)90003-7

[22] Hasen NS, Gammie SC (2009) Trpc2 gene impacts on maternal aggression, accessory olfactory bulb anatomy and brain activity. Genes Brain Behav 8: 639–649. 19799641 10.1111/j.1601-183X.2009.00511.x PMC2758541

[23] Wang Z, Storm DR (2011) Maternal behavior is impaired in female mice lacking type 3 adenylyl cyclase. Neuropsychopharmacology 36: 772–781. 21150908 10.1038/npp.2010.211 PMC3055720

[24] Jyotika J, McCutcheon J, Laroche J, Blaustein JD, Forger NG (2007) Deletion of the Bax gene disrupts sexual behavior and modestly impairs motor function in mice. Dev Neurobiol 67: 1511–1519. 17525992 10.1002/dneu.20525

[25] Wu MV, Manoli DS, Fraser EJ, Coats JK, Tollkuhn J, et al (2009) Estrogen masculinizes neural pathways and sex-specific behaviors. Cell 139: 61–72. 19804754 10.1016/j.cell.2009.07.036 PMC2851224

[26] Kimchi T, Xu J, Dulac C (2007) A functional circuit underlying male sexual behaviour in the female mouse brain. Nature 448: 1009–1014. 17676034 10.1038/nature06089

[27] Spors H, Sobel N (2007) Male behavior by knockout. Neuron 55: 689–693. 17785177 10.1016/j.neuron.2007.08.016

[28] Haga S, Hattori T, Sato T, Sato K, Matsuda S, et al (2010) The male mouse pheromone ESP1 enhances female sexual receptive behaviour through a specific vomeronasal receptor. Nature 466: 118–122. 20596023 10.1038/nature09142

[29] Bean NJ, Wysocki CJ (1989) Vomeronasal organ removal and female mouse aggression: the role of experience. Physiol Behav 45: 875–882. 2780872 10.1016/0031-9384(89)90209-6

[30] Hasen NS, Gammie SC (2011) Trpc2-deficient lactating mice exhibit altered brain and behavioral responses to bedding stimuli. Behav Brain Res 217: 347–353. 21070815 10.1016/j.bbr.2010.11.002 PMC3010422

[31] Kingston PA, Zufall F, Barnstable CJ (1999) Widespread expression of olfactory cyclic nucleotide-gated channel genes in rat brain: implications for neuronal signalling. Synap N Y N 32: 1–12. 10.1002/(SICI)1098-2396(199904)32:1<1::AID-SYN1>3.0.CO;2-M 10188632

[32] Edwards DA, Burge KG (1973) Olfactory control of the sexual behavior of male and female mice. Physiol Behav 11: 867–872. 4763476 10.1016/0031-9384(73)90282-5

[33] Belluscio L, Gold GH, Nemes A, Axel R (1998) Mice deficient in G(olf) are anosmic. Neuron 20: 69–81. 9459443 10.1016/s0896-6273(00)80435-3

[34] Wang Z, Li V, Chan GCK, Phan T, Nudelman AS, et al (2009) Adult type 3 adenylyl cyclase-deficient mice are obese. PloS One 4: e6979. 19750222 10.1371/journal.pone.0006979 PMC2735775

[35] Lin W, Arellano J, Slotnick B, Restrepo D (2004) Odors Detected by Mice Deficient in Cyclic Nucleotide-Gated Channel Subunit A2 Stimulate the Main Olfactory System. J Neurosci 24: 3703–3710. 15071119 10.1523/JNEUROSCI.0188-04.2004 PMC6729751

[36] Leinders-Zufall T, Cockerham RE, Michalakis S, Biel M, Garbers DL, et al (2007) Contribution of the receptor guanylyl cyclase GC-D to chemosensory function in the olfactory epithelium. Proc Natl Acad Sci 104: 14507–14512. 17724338 10.1073/pnas.0704965104 PMC1964822

[37] Kelliher KR, Spehr M, Li X-H, Zufall F, Leinders-Zufall T (2006) Pheromonal recognition memory induced by TRPC2-independent vomeronasal sensing. Eur J Neurosci 23: 3385–3390. 16820028 10.1111/j.1460-9568.2006.04866.x

[38] Kim S, Ma L, Jensen KL, Kim MM, Bond CT, et al (2012) Paradoxical contribution of SK3 and GIRK channels to the activation of mouse vomeronasal organ. Nat Neurosci 15: 1236–1244. 22842147 10.1038/nn.3173 PMC3431453

