# Supplementary figures and images for: Complex Chemosensory Control of Female Reproductive Behaviors
Source: PLoS One. 2014 Feb 28;9(2):e90368. doi: 10.1371/journal.pone.0090368 (PMC3938725; doi:10.1371/journal.pone.0090368)

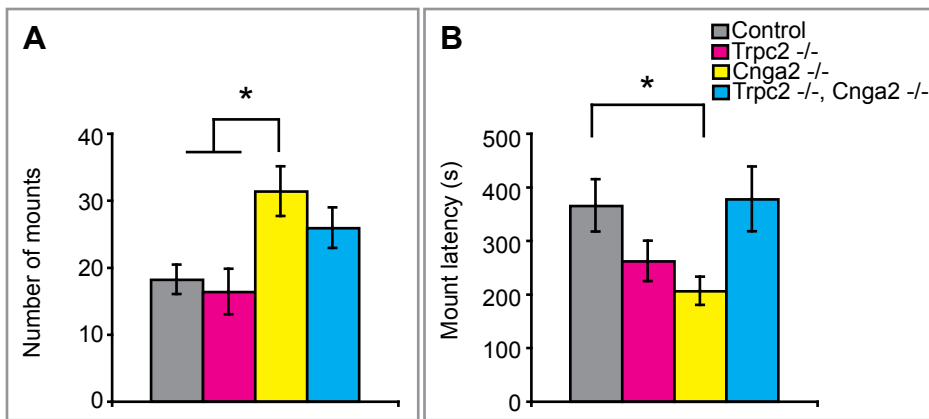

Supplement: Figure S1 — Decreased sexual receptivity of Cnga2 or Trpc2 null females is not a consequence of reduced mounting by WT males. (A) WT males mount all females ≥10 mounts/assay on average. Cnga2 null females are mounted more times than Trpc2 null or control females. Kruskal-Wallis: χ2(3,56) = 13.5, p = 0.007. (B) WT males mount all females within the first 400 s of an assay on average, which lasts for 1800 s. Cnga2 null females are mounted faster than control females. One-Way ANOVA: F(3,56) = 3.08, p = 0.035. Mean ± SEM; *p<0.05; N≥8/cohort. (PDF) [file pone.0090368.s001.pdf]

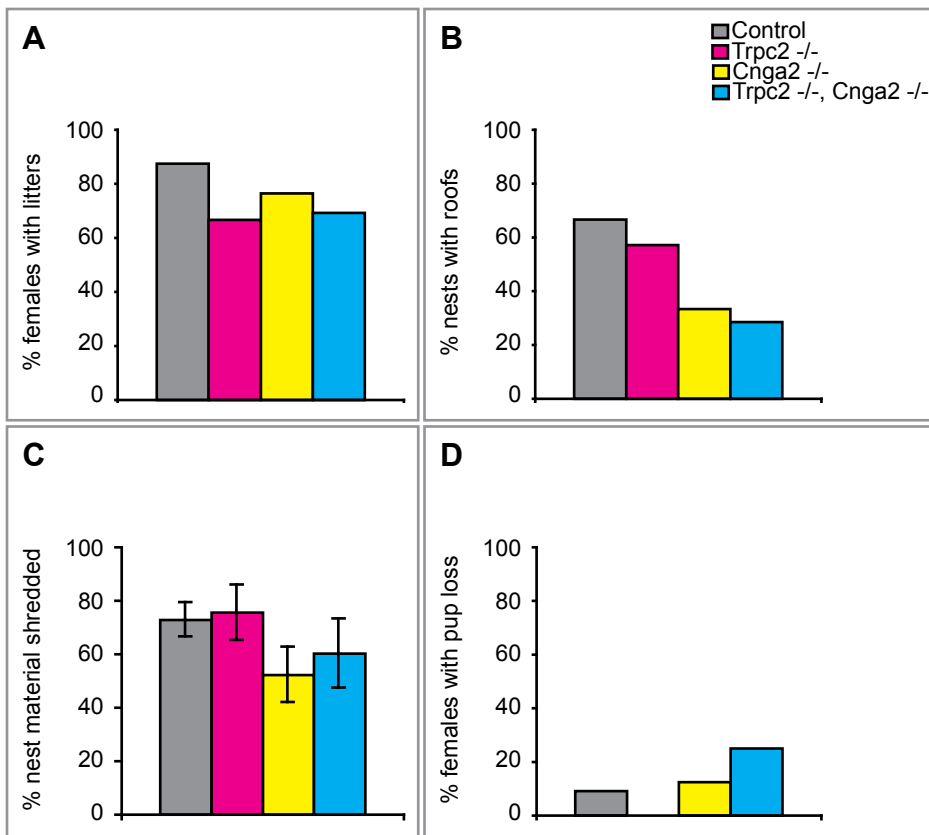

Supplement: Figure S2 — Cnga2 and Trpc2 are not required for fertility, litter survival, or nest building. (A) When co-housed with a male for at least 1 week, females of all genotypes produced litters within 26 days of co-housing. (B) Females of all genotypes built nests of equivalent quality as assessed by the presence of walls and a roof. (C) Females of all genotypes utilized comparable amount of nest material to construct their nests. (D) Comparable survival of litters delivered to females of all genotypes. Mean ± SEM. N≥7/cohort. (PDF) [file pone.0090368.s002.pdf]

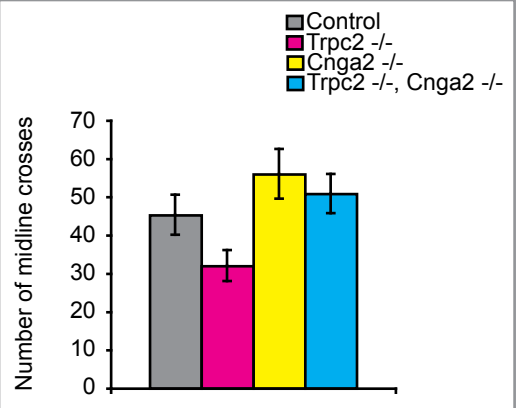

Supplement: Figure S3 — Failure to retrieve pups in Cnga2-/- and Cnga2-/- , Trpc2-/- dams is not due to decreased locomotor activity. Number of midline crosses during pup retrieval assays in olfactory mutants is comparable to controls. Mean ± SEM. N≥5/cohort. (PDF) [file pone.0090368.s003.pdf]
